# The number of metabolic syndrome risk factors predicts alterations in gut microbiota in Chinese children from the Huantai study

**DOI:** 10.1186/s12887-023-04017-x

**Published:** 2023-04-21

**Authors:** Jiahong Sun, Xiaoyun Ma, Liu Yang, Xuli Jin, Min Zhao, Bo Xi, Suhang Song

**Affiliations:** 1 Department of Epidemiology, School of Public Health, Cheeloo College of Medicine, Shandong University, 44 Wen Hua Xi Road, Jinan, 250012 China; 2 Department of Nutrition and Food Hygiene, School of Public Health, Cheeloo College of Medicine, Shandong University, Jinan, Shandong China; 3 Taub Institute for Research in Alzheimer ’s disease and the Aging Brain, Columbia University, New York, NY USA

**Keywords:** Metabolic syndrome, Children, Gut microbiota, 16S rRNA

## Abstract

**Background:**

Evidence on the effect of gut microbiota on the number of metabolic syndrome (MetS) risk factors among children is scarce. We aimed to examine the alterations of gut microbiota with different numbers of MetS risk factors among children.

**Methods:**

Data were collected from a nested case–control study at the baseline of the Huantai Childhood Cardiovascular Health Cohort Study in Zibo, China. We compared the differences in gut microbiota based on 16S rRNA gene sequencing among 72 children with different numbers of MetS risk factors matched by age and sex (i.e., none, one, and two-or-more MetS risk factors; 24 children for each group).

**Results:**

The community richness (i.e., the total number of species in the community) and diversity (i.e., the richness and evenness of species in the community) of gut microbiota decreased with an increased number of MetS risk factors in children (*P* for trend < 0.05). Among genera with a relative abundance greater than 0.01%, the relative abundance of *Lachnoclostridium* (*P*_*FDR*_ = 0.009) increased in the MetS risk groups, whereas *Alistipes* (*P*_*FDR*_ < 0.001) and *Lachnospiraceae_NK4A136_grou*p (*P*_*FDR*_ = 0.043) decreased in the MetS risk groups compared to the non-risk group. The genus *Christensenellaceae_R-7_group* excelled at distinguishing one and two-or-more risk groups from the non-risk group (area under the ROC curve [AUC]: 0.84 − 0.92), while the genera *Family_XIII_AD3011_group* (AUC: 0.73 − 0.91) and *Lachnoclostridium* (AUC: 0.77 − 0.80) performed moderate abilities in identifying none, one, and two-or-more MetS risk factors in children.

**Conclusions:**

Based on the nested case–control study and the 16S rRNA gene sequencing technology, we found that dysbiosis of gut microbiota, particularly for the genera *Christensenellaceae_R-7_group*, *Family_XIII_AD3011_group*, and *Lachnoclostridium* may contribute to the early detection and the accumulation of MetS risk factors in childhood.

**Supplementary Information:**

The online version contains supplementary material available at 10.1186/s12887-023-04017-x.

## Background

Metabolic syndrome (MetS) has attracted widespread attention due to its association with cardiovascular disease (CVD), which is a major contributor to the burden of disability and the leading cause of death worldwide [1–3]. MetS is a complex disorder with several risk factors including abdominal obesity, dyslipidemia (increased triglyceride [TG] and decreased high-density lipoprotein cholesterol [HDL-C]), elevated blood pressure (BP), and hyperglycemia [4]. It has been reported that the prevalence of individual and clustering of MetS risk factors among children and adolescents has been alarmingly increasing in recent years [5, 6]. In addition, the number of MetS risk factors was associated with gradually increasing odds of short-term and long-term cardiovascular damage [7, 8]. Therefore, early detection of the accumulation of MetS risk factors is important for the prevention of CVD and related morbidity later in life.

Recently, microbiome-based interventions have been gaining popularity to treat and prevent metabolic disorders. Previous studies based on mice models and adults showed the association of alteration of gut microbiota with MetS and its risk factors [9–12]. However, limited studies have paid attention to the association in children. The gut microbiota of children has been shown to be more susceptible to environmental factors than that of adults, and thus the gut microbiota associated with adult MetS cannot be generalized to children [13]. Several studies have investigated the effect of the change in gut microbiota on individual MetS risk factor (e.g., obesity, elevated BP, hyperglycemia) among children [14–18]. However, to the best of our knowledge, little is known about the gut microbiota in identifying the number of MetS risk factors among children. Clarifying this association may open avenues for convenient prevention, diagnosis, and treatment of the clustering of MetS risk factors in childhood and thus reduce the huge burden of CVD in adulthood.

Therefore, in this study, we aimed to identify the differential gut microbiota among children aged 10 − 11 years with none, one, and two or more numbers of MetS risk factors.

## Methods

### Participants and sample collection

This was a nested case–control study from the baseline of the “Huantai Childhood Cardiovascular Health Cohort Study” including 1515 children aged 6 − 11 years old, among whom we identified 24 children with two or more MetS risk factors. To control for confounding factors such as age and sex, we conducted a 1:1:1 propensity score matching to select 24 children without MetS risk factors and 24 children with one MetS risk factor (none MetS risk factor: 10.83 ± 0.35 years old; one MetS risk factor: 10.73 ± 0.32 years old; two-or-more MetS risk factors: 10.72 ± 0.33 years old; male: female = 15:9 in each group). Thus, a total of 72 children aged 10 − 11 years without the use of antibiotics and probiotics in the past three months were included in this study (24 children without MetS risk factors, 24 with one risk, and 24 with two or more risks). All included children were without a history of gastrointestinal disorders or diarrhea and were not taking medications at the time of the study. The informed consent was written by all participants and their guardians. This study was approved by the Ethics Committee of Shandong University.

### Clinical data collection

Anthropometrics (e.g., weight, height, BP, and waist circumference [WC]), demographic characteristics (e.g., age and sex), and blood biochemistry indexes (e.g., fasting plasma glucose [FPG], TG, HDL-C, and low-density lipoprotein cholesterol [LDL-C]) were collected in this study. Specifically, height and weight were measured twice in light clothes without shoes using an ultrasonic height and weight scale (Shengyuan Co. Ltd, HGM-300, Henan, China). The mean values of two height and weight measurements were used for data analyses with an accuracy of 0.1 cm and 0.1 kg for height and weight, respectively [19]. Body mass index (BMI) was calculated by weight (kg) divided by height (m) squared. WC was measured twice using a non-elastic measuring tape at 1 cm above the navel around a week horizontally, and the mean values of two WC measurements were used for data analyses with an accuracy of 0.1 cm [19]. BP was measured three times continuously with the deviation of any two BP values controlled within 4 mmHg (OMRON-HEM 7012, Osaka, Japan), and the mean values of three BP measurements were used for data analyses [20]. FPG, TG, HDL-C, and LDL-C were measured using a Beckman Coulter AU480 automatic analyzer (Mishima, Shizuoka, Japan) [20].

### Definition

Children received one point for each MetS risk factor if they met the criteria outlined as follows: (1) elevated BP: systolic BP (SBP) and/or diastolic BP (DBP) ≥ the age- and sex-specific 90^th^ percentile [21]; (2) hyperglycemia: FPG ≥ 5.6 mmol/L [22]; (3) dyslipidemia: TG ≥ 1.47 mmol/L; (4) dyslipidemia: HDL-C ≤ 1.03 mmol/L [23]; (5) abdominal obesity: WC ≥ the age- and sex-specific 90^th^ percentile [24]. Thus, based on the number of MetS risk factors, children were classified into three groups (non-risk, one-risk, and two-or-more-risks). In this study, alterations of gut microbiota refer to the difference in the composition and relative abundance of dominant species, community richness (i.e., the total number of species in the community) and diversity (i.e., the richness and evenness of species in the community), and differential species among groups with an increasing number of MetS risk factors compared with the group without MetS risk factors.

### Basic characteristics

The frequency of fruit and vegetable intake each day, the frequency of soft drink intake every week, and physical activity were classified into < 3 times/day vs. ≥ 3 times/day, < 3 times/week vs. ≥ 3 times/week, and < 1 h/day vs. ≥ 1 h/day, respectively. Parental education was divided into lower than high school and high school or higher (i.e., one of parents with high school or higher). Both paternal smoking and drinking were classified as yes and no.

### Fecal samples collection and processing

Fresh fecal samples from children who had not received antibiotics within the past three months were collected in sterile fecal tubes and then frozen in − 80 °C refrigerators. Microbial genomic DNA (gDNA) was extracted from the fecal samples and detected by 1% agarose gel electrophoresis [25]. 16S rRNA gene was selected as bacterial specific fragment using 338F (5'-ACTCCTACGGGAGGCAGCAG-3') and 806R (5'-GGACTACHVGGGTWTCTAAT-3') primers [26]. Amplifications were performed using TransGen AP221-02: TransStart Fastpfu DNA Polymerase [27]. A two-stage PCR was performed in the ABI GeneAmp® 9700 (Applied Biosystems Inc. USA) in triplicate [28]. Then, the PCR products of the same sample were mixed and detected using 2% agarose gel electrophoresis, followed by recovering with AxyPrepDNA Gel Recovery Kit (Axygen; Corning, Inc., Corning, NY, USA) and eluting with Tris–HCl [29].

According to the preliminary quantitative results of electrophoresis, the PCR products were detected and quantified with the QuantiFluor™-ST Blue Fluorescence Quantitative System (Promega Corp., Madison, WI, USA) [30]. The purified amplicons were pooled in equimolar amounts and sequenced on an Illumina Hiseq3000 platform (Illumina, SanDiego, CA, USA) according to the standard protocols.

### Sequence processing and analysis

Quality control of the raw sequencing reads was performed using the FastQC tool (https://www.bioinformatics.babraham.ac.uk/projects/fastqc/) [31] to filter the bad reads, the low-quality bases, adapters, and N-bases [32]. According to the overlap relation between Pair-end (PE) reads, we merged pairs of reads into a sequence with a minimum length of overlap of 10 bp [33]. After detecting and filtering the chimera sequence, the data were analyzed with the Quantitative Insights Into Microbial Ecology (QIIME 1.9.1; http://qiime.org/install/index.html) toolkit to obtain the optimization sequence [34]. The raw sequencing reads have been submitted to the Sequence Read Archive (SRA) of the National Center for Biotechnology Information (NCBI) database (BioProject ID: PRJNA775883; https://www.ncbi.nlm.nih.gov/bioproject/PRJNA775883), and this deposited data is available in the NCBI database.

According to a 97% similarity cut-off, the Operational Taxonomic Units (OTU) clustering was performed for non-repeating sequences (excluding single sequences) using Uparse software (version 7.0.1090; http://drive5.com/uparse/). At the same time, chimeric sequences were identified and removed to obtain representative sequences of OTUs [29]. According to the Silva database (Release 138; http://www.arb-silva.de), taxonomic annotation was performed on the OTUs representative sequences of each sample based on the RDP classifier Bayesian algorithm Classifier (version 2.11; http://sourceforge.net/projects/rdp-classifier/) with a 0.7 confidence threshold [29].

### Statistical analyses

The continuous variables were presented as mean and standard deviation (SD), and the categorical variables were presented as n (%). The Analysis of Variance was performed to compare the differences in age, WC, BMI, SBP, DBP, FPG, TG, LDL-C, and HDL-C among the three groups, and the Chi-square test was performed to compare the differences in sex, the frequency of fruit and vegetable intake each day, the frequency of soft drink intake every week, physical activity, parental education, paternal smoking, paternal drinking, parental BMI, parental history of hypertension, heart disease, stroke, and diabetes among the three groups. SPSS 25.0 software (IBM, Armonk, NY, USA) and R language (version 3.3.1) were used for analysis. Two-sided *P* values < 0.05 indicate a significant difference.

The rarefaction curve was used to explore the sequencing depth as well as the abundance of sample species with different sequencing quantities based on the Sobs index (community richness) and Shannon index (community diversity). The Venn diagram analysis was performed to count the number of common and unique OTUs among the three groups. Among genera with a relative abundance greater than 0.01%, the bar plot and the non-parametric Kruskal–Wallis H test were used to compare the changes in composition and relative abundance of genera among the three groups with the adjustment of the false discovery rate (FDR).

The *α*-diversity indexes including the Ace, Chao 1, Shannon, and Inverse Simpson were calculated to evaluate the community diversity and richness of gut microbiota at the OTU level using the Mothur software platform (version 1.30.2; https://www.mothur.org/wiki/Download_mothur) [35]. A trend test was used to estimate trends in *P* values for the *α*-diversity indexes of gut microbiota and the number of MetS risk factors. The difference in *β*-diversity among the three groups was calculated according to the Bray–Curtis distance matrix using permutational ANOSIM in the Principal Coordinate Analysis (PCoA) and Non-metric multidimensional scaling analysis (NMDS) [36].

At the genus level, the Linear Discriminant Analysis Effect Size (LEfSe) and the linear discriminant analyses (LDA) were performed to evaluate the extent of the contribution of differential gut microbiota to the different numbers of MetS risk factors. The random forest model analysis was conducted to screen out the top ten genera biomarkers to distinguish the groups with at least one MetS risk group from the non-risk group based on the randomForest package of the R language. We further evaluate the differences in the relative abundance of these top ten genera biomarkers among the three groups based on the non-parametric Kruskal–Wallis H test with the FDR. Finally, the significant genera in the LEfSe analysis, the random forest analyses, and the non-parametric Kruskal–Wallis H test were selected as potential diagnostic biomarkers for the different numbers of MetS risk factors in children. The receiver operating characteristic curve (ROC) was performed to evaluate the ability of these selected genera in identifying the number of MetS risk factors (i.e., area under the ROC curve [AUC]) based on the pROC package of the R language.

Phylogenetic Investigation of Communities by Reconstruction of Unobserved States 2 (PICRUSt2) software was performed to predict the Kyoto Encyclopedia of Genes and Genomes (KEGG) pathways of gut microbiota based on the Greengene database [37]. The non-parametric Kruskal–Wallis H test was performed to compare the differences in KEGG level 3 pathways among the three groups. Then, the Post-hoc test with Tukey Kramer was used for further pairwise comparisons on the differential pathways based on the STAMP software [38] (Additional file 1).

## Results

### Study population

A total of 72 children aged 10.8 ± 0.3 years were included in this study and were categorized into three groups, including the non-risk group (*n* = 24), the one-risk group (*n* = 24), and the two-or-more-risk group (*n* = 24). The number of children with different MetS risk factors in each group is presented in Table S1. As shown in Table 1, no significant differences were observed in age, sex, FPG, LDL-C, the frequency of fruit and vegetable intake each day, the frequency of soft drink intake every week, parental education, paternal smoking, paternal drinking, and physical activity among these three groups. Compared with the non-risk group, the other two groups with at least one MetS risk factor reported a significantly higher WC (*P* < 0.001), BMI (*P* < 0.001), SBP (*P* < 0.001), DBP (*P* < 0.001), and TG (*P* < 0.001), and a marginally lower HDL-C (*P* = 0.052). Moreover, there were no significant differences in parental BMI, parental history of hypertension, heart disease, stroke, and diabetes across the three groups (Table S2).

**Table 1:** Characteristics of children among three groups

Parameters and clinical data	Non-risk (*n* = 24)	One-risk (*n* = 24)	Two-or-more-risks (*n* = 24)	*F/χ*^*2*^	*P*-value
Age (years; mean ± sd)	10.83 ± 0.35	10.73 ± 0.32	10.72 ± 0.33	0.72^a^	0.492
Sex (male, n %)	15 (62.5%)	15 (62.5%)	15 (62.5%)	0.00^b^	1.000
WC (cm; mean ± sd)	60.71 ± 3.34	72.09 ± 11.31	82.35 ± 9.40	37.09^a^	< 0.001
BMI (kg/m^2^; mean ± sd)	16.28 ± 1.44	21.30 ± 4.00	25.41 ± 3.70	47.39^a^	< 0.001
SBP (mmHg; mean ± sd)	104.06 ± 6.00	111.77 ± 7.48	117.54 ± 6.94	23.51^a^	< 0.001
DBP (mmHg; mean ± sd)	60.98 ± 4.19	64.48 ± 5.66	68.02 ± 6.73	9.41^a^	< 0.001
FPG (mmol/L; mean ± sd)	4.61 ± 1.08	4.43 ± 1.77	4.81 ± 1.24	0.46^a^	0.636
TG (mmol/L; mean ± sd)	0.75 ± 0.29	0.97 ± 0.47	1.26 ± 0.46	9.14^a^	< 0.001
HDL-C (mmol/L; mean ± sd)	1.70 ± 0.54	1.41 ± 0.71	1.31 ± 0.41	3.09^a^	0.052
LDL-C (mmol/L; mean ± sd)	2.16 ± 0.79	2.08 ± 1.00	2.52 ± 0.85	1.68^a^	0.194
Fruit-vegetables (n %)				0.79^b^	0.673
< 3 times/day	12 (50.0%)	15 (62.5%)	14 (58.3%)		
≥ 3 times/day	12 (50.0%)	9 (37.5%)	10 (41.7%)		
Soft drink (n %)				2.69^c^	0.351
< 3 times/week	23 (95.8%)	23 (95.8%)	20 (83.3%)		
≥ 3 times/week	1 (4.2%)	1 (4.2%)	4 (16.7%)		
Parental education (n %)				1.52^c^	0.469
Lower than high school	4 (16.7%)	7 (29.2%)	4 (16.7%)		
High school or higher	20 (83.3%)	17 (70. 8%)	20 (83.3%)		
Paternal smoking (n %)				0.84^b^	0.656
No	10 (41.7%)	7 (29.2%)	9 (37.5%)		
Yes	14 (58.3%)	17 (70.8%)	15 (62.5%)		
Paternal drinking (n %)				0.00^c^	1.000
No	2 (8.3%)	2 (8.3%)	2 (8.3%)		
Yes	22 (91.7%)	22 (91.7%)	32 (91.7%)		
Physical activity (n %)				1.37^b^	0.504
< 1 h/day	12 (50.0%)	16 (66.7%)	14 (58.3%)		
≥ 1 h/day	12 (50.0%)	8 (33.3%)	10 (41.7%)		

*Note*: ^a^Analysis of Variance; ^b^chi-square test; ^c^Fisher’s exact test; *sd* standard deviation, *WC* waist circumference, *BMI* body mass index, *sd* standard deviation, *SBP* systolic blood pressure, *DBP* diastolic blood pressure, *FPG* fasting plasma glucose, *TG* triglyceride, *HDL-C* high-density lipoprotein cholesterol, *LDL-C* low-density lipoprotein cholesterol

### OTU analysis among the three groups

A total of 1,026 OTUs were obtained. In the Venn diagram, 590 OTUs were shared by three groups, and 144, 47, and 30 unique OTUs were observed in the non-risk, one-risk, and two-or-more-risk groups, respectively (Fig. S1a). The rarefaction curves of the Shannon and Sobs index revealed that the sequencing data met the requirements (i.e., the sample size of participants and sampling depth were reasonable) of the analysis (Fig. S1b, c).

### Differences in community diversity and richness of gut microbiota

**Fig. 1:**
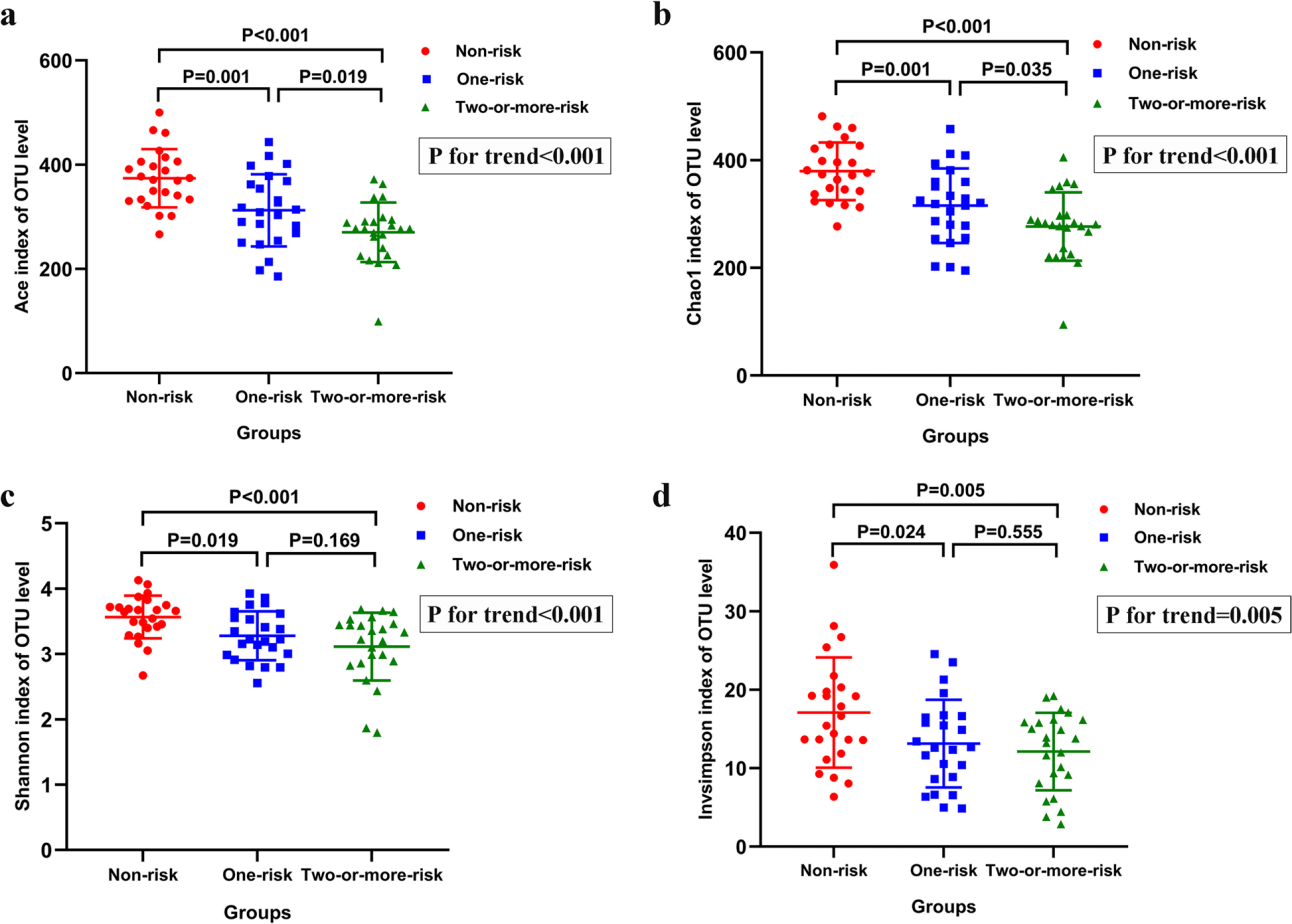
Differences in *α*-diversity of gut microbiota among the three groups. **(a)** Ace index, **(b)** Chao 1 index, **(c)** Shannon index, and **(d)** Inverse Simpson

The results of *α*-diversity analyses showed that the community richness indexes including Ace index (one vs. none: *P* = 0.001; two-or-more vs. none: *P* < 0.001; and two-or-more vs. one: *P* = 0.019, Fig. 1a) and Chao 1 index (one vs. none: *P* = 0.001; two-or-more vs. none: *P* < 0.001; and two-or-more vs. one: *P* = 0.035, Fig. 1b) were highest in the non-risk group, followed by the one-risk and two-or-more risk groups. The community diversity indexes, including the Shannon index (one vs. none: *P* = 0.019; two-or-more vs. none: *P* < 0.001, Fig. 1c) and the Inverse Simpson index (one vs. none: *P* = 0.024; two-or-more vs. none: *P* = 0.005, Fig. 1d), were only statistically different between the non-risk group and the one-risk group or two-or-more-risk group. The trend analyses showed that all the Ace index, Chao 1 index, Shannon index, and Inverse Simpson index decreased with the increased number of MetS risk factors (all *P* for trends < 0.01).

### *β*-diversity analysis of gut microbiota among the three groups

The PCoA analysis indicated that the gut microbiota across the three MetS risk factor groups could be statistically separated (*P* = 0.006; Fig. S2a). Additionally, NMDS analysis showed that the composition of gut microbiota was statistically separated among three MetS risk factor groups (*P* = 0.006; Fig. S2b).

### Differences in the composition and relative abundance of gut microbiota among the three groups

**Fig. 2:**
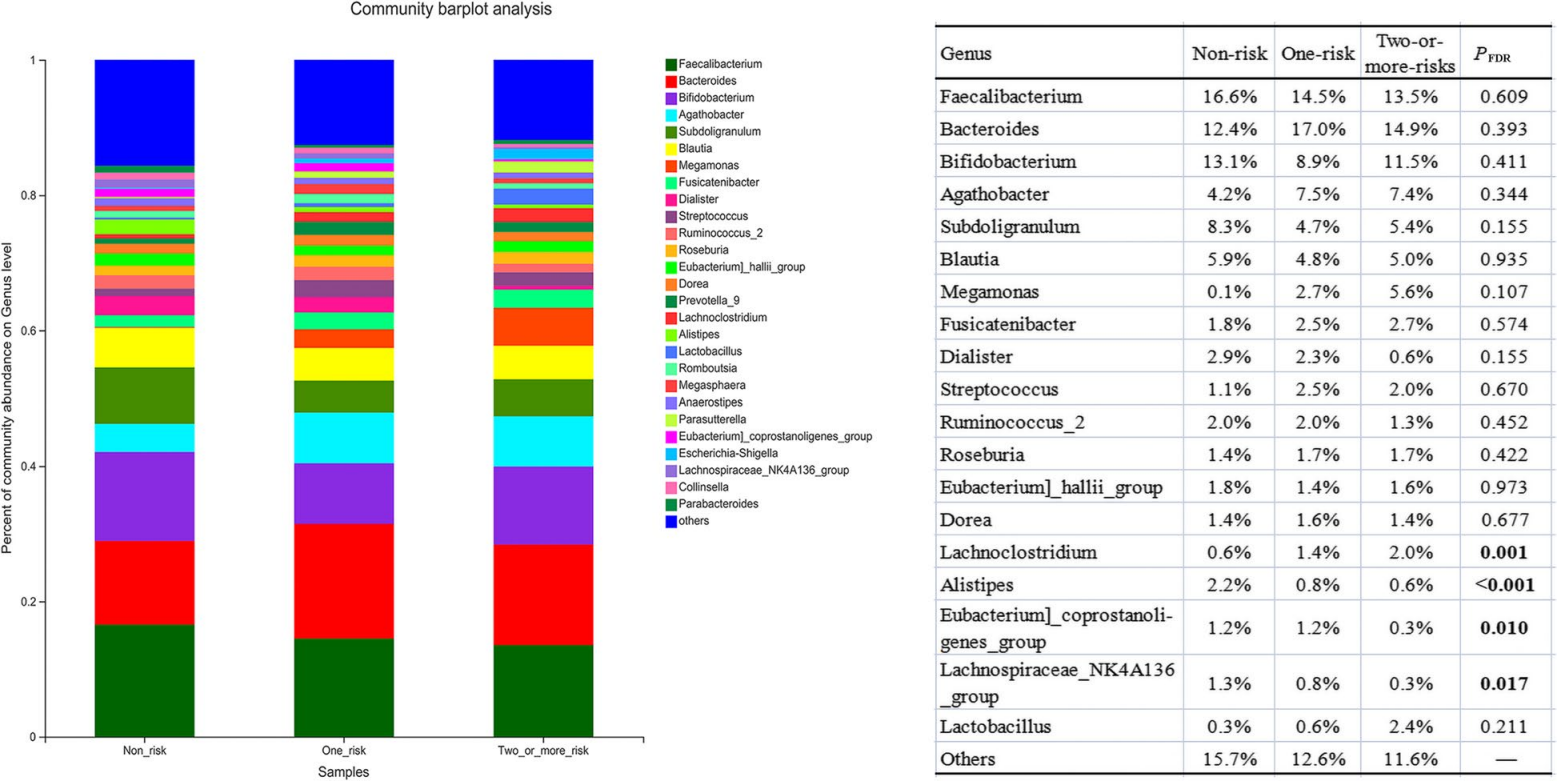
Differences in the composition and relative abundance of gut microbiota at the genus level among the three groups based on the non-parametric Kruskal–Wallis H test

Among genera with a relative abundance greater than 0.01%, the relative abundance of *Faecalibacterium*, *Bifidobacterium*, *Bacteroides*, and *Subdoligranulum* ranked the top four in the non-risk group, while *Bacteroides*, *Faecalibacterium*, *Bifidobacterium*, and *Agathobacter* ranked the top four in both the one-risk group and two-or-more-risk group. In addition, the relative abundance of *Lachnoclostridium* (*P*_*FDR*_ = 0.001) significantly increased in the one-risk group and two-or-more-risk group, while the relative abundance of *Alistipes* (*P*_*FDR*_ < 0.001) and *Lachnospiraceae_NK4A136_group* (*P*_*FDR*_ = 0.017) decreased in the one-risk group and two-or-more-risk group compared with the non-risk group (Fig. 2).

### Screening out biomarkers associated with the number of MetS risk factors

Under the LDA threshold of 2.0, LEfSe and LDA analyses showed that a total of 33, 4, and 4 genera were enriched in the non-risk group, one-risk group, and two-or-more-risk group, respectively (Fig. S3a and Table S3). The random forest analysis found that *Christensenellaceae_R-7_group*, *Family_XIII_AD3011_group*, *Ruminiclostridium_6*, *Tyzzerella_4*, *Ruminococcaceae_UCG-002*, *Lachnoclostridium*, *unclassified_o__Bacteroidales*, *Lachnospira*, *Parasutterella*, and *Ruminococcaceae_UCG-005* were the top ten biomarkers associated with the different numbers of MetS risk factors at the genus level (Fig. S3b and Table S4), and these ten genera were also significant in the LEfSe and LDA analyses. Furthermore, we found *Lachnoclostridium* (*P*_*FDR*_ = 0.009) was significantly enriched in the one-risk group and two-or-more-risk group, whereas *Ruminococcaceae_UCG-002* (*P*_*FDR*_ = 0.002), *Christensenellaceae_R-7_group* (*P*_*FDR*_ < 0.001), *Ruminococcaceae_UCG-005* (*P*_*FDR*_ = 0.009), *Tyzzerella_4* (*P*_*FDR*_ = 0.020), *Family_XIII_AD3011_group* (*P*_*FDR*_ < 0.001), *Ruminiclostridium_6* (*P*_*FDR*_ = 0.002), and *unclassified_o__Bacteroidales* (*P*_*FDR*_ < 0.001) were significantly enriched in the non-risk group based on the non-parametric Kruskal–Wallis H test (Fig. S3c and Table 2).

**Table 2:** The mean relative abundance of gut microbiota biomarkers based on the non-parametric Kruskal–Wallis H test

Feature	Non-risk (*n* = 24)	One-risk (*n* = 24)	Two-or-more-risks (*n* = 24)	*P*_FDR_
*Christensenellaceae_R-7_group*	0.83	0.17	0.10	< 0.001
*Family_XIII_AD3011_group*	0.06	0.02	0.01	< 0.001
*Ruminiclostridium_6*	0.04	0.003	0.002	0.002
*Tyzzerella_4*	0.07	0.04	0.01	0.020
*Ruminococcaceae_UCG-002*	0.67	0.36	0.13	0.002
*Lachnoclostridium*	0.60	1.43	2.03	0.009
*Unclassified_o__Bacteroidales*	0.02	0.003	0.004	< 0.001
*Ruminococcaceae_UCG-005*	0.24	0.08	0.02	0.009

### Exploring the diagnostic efficacy of potential biomarkers

**Fig. 3:**
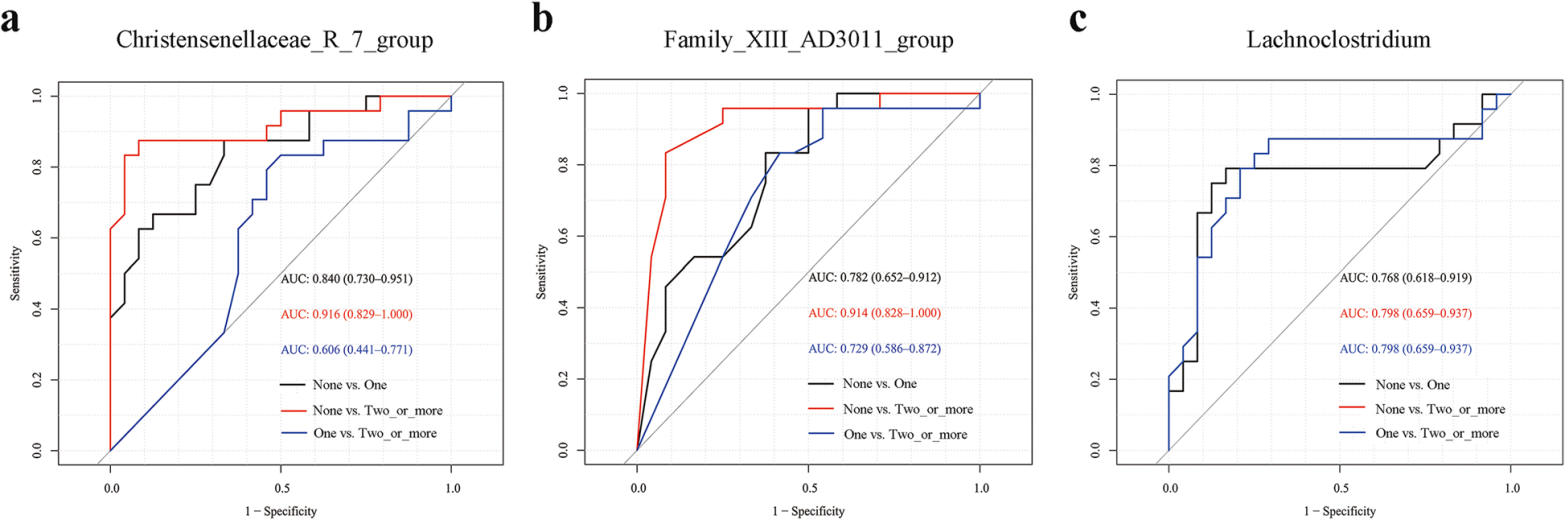
Evaluating the diagnostic efficacy of gut microbiota biomarkers associated with MetS risk factors among children. **(a)** *Christensenellaceae_R-7_group*, **(b)** *Family_XIII_AD3011_group*, and **(c)** *Lachnoclostridium* (The red curve is the same as the blue curve)

We used ROC analyses to explore the diagnostic efficacy of these eight genera and found that *Christensenellaceae_R-7_group* had a higher ability in distinguishing the one-risk group (AUC = 0.84, 95% CI: 0.73 − 0.95, *P* < 0.001) and two-or-more-risk group (AUC = 0.92, 95% CI: 0.83 − 1.00, *P* < 0.001) from the non-risk group, while this genus had a weak ability to distinguish two-or-more-risk group from one-risk group (AUC = 0.61, 95% CI: 0.44 − 0.77, *P* > 0.050; Fig. 3a). Besides, *Family_XIII_AD3011_group* similarly had a powerful capacity to differentiate the two-or-more-risk group (AUC = 0.91, 95% CI: 0.83 − 1.00, *P* < 0.001) from the non-risk group, and a moderate capacity to differentiate the one-risk group from the non-risk group (AUC = 0.78, 95% CI: 0.65 − 0.91, *P* < 0.050), as well as the two-or-more-risk group from the one-risk group (AUC = 0.73, 95% CI: 0.59 − 0.87, *P* < 0.050; Fig. 3b). In addition, *Lachnoclostridium* had a moderate ability to differentiate these three groups (AUC > 0.77, *P* < 0.05) (Fig. 3c, the ROC for two-or-more vs. none risk and two-or-more vs. one risk are overlapped). The ROC results of the other five genera are presented in Fig. S1d-h.

### Differences in KEGG pathways among the three groups

The KEGG pathway analyses showed that D-Glutamine and D-glutamate metabolism, cysteine and methionine metabolism, polyketide sugar unit biosynthesis, cationic antimicrobial peptide (CAMP) resistance, and acarbose and validamycin biosynthesis were the differential pathways among the three groups (all *P* < 0.001). Except for the cationic antimicrobial peptide (CAMP) resistance pathway enriched in the MetS risk groups, the other four pathways were all enriched in the non-risk group (Fig. S4).

## Discussion

To the best of our knowledge, we initially found that the dysbiosis of gut microbiota was associated with the different numbers of MetS risk factors among children. There was a downward trend in the community diversity and richness of gut microbiota with the increased number of MetS risk factors. Among genera with a relative abundance greater than 0.01%, the genus *Lachnoclostridium* increased in the MetS risk groups, whereas the genera *Alistipes* and *Lachnospiraceae_NK4A136_group* decreased in the MetS risk groups compared with the non-risk group. In addition, the genera *Christensenellaceae_R-7_group*, *Family_XIII_AD3011_group*, and *Lachnoclostridium* performed moderately well in identifying the number of MetS risk factors. Our KEGG pathway analyses showed that D-Glutamine and D-glutamate metabolism and cysteine and methionine metabolism pathways might participate in cardiovascular homeostasis through the regulation of gut microbiota. These findings confirmed that gut microbiota played a pivotal part in identifying the number of MetS risk factors among Chinese children.

It has been shown that specific MetS risk factors are inversely associated with the community richness and diversity of gut microbiota among adults [39]. For example, individuals with obesity, dyslipidemia, or hypertension were more likely to obtain a lower community richness of gut microbiota compared with normal controls [40–42]. In addition, previous studies found that increased BMI and blood lipid levels (e.g., TG) were associated with reduced bacterial diversity among adults [42, 43]. Alterations in the composition of gut microbiota have also been reported to be linked to atherosclerosis, hypertension, obesity, and type 2 diabetes mellitus [39]. However, these previous findings only focused on one MetS risk factor or were limited to adults, ignoring the association between the clustering of MetS risk factors and the imbalance of gut microbiota. In this study, we not only found that the richness estimators of gut microbiota decreased among children with one MetS risk factor, but also further found that there was a decreasing trend of community diversity and richness with the accumulation of MetS risk factors among children.

There were complex interactive effects of genetic background, gut microbiota, and diet on the development of obesity and MetS features [44]. In this study, we found that there are no differences in diet across these three groups, suggesting that the differential gut microbiota across groups might be due to genetic influences. However, the diet information we collected was self-reported, which might bias the true results. Besides, we additionally performed analyses on the difference in parental BMI, and parental history of hypertension, heart disease, stroke, and diabetes across three groups and found that there were no differences across three groups. Our findings suggest that other unmeasured variables might affect the association between gut microbiota and the number of MetS risk factors. Future studies with larger sample sizes, more accurate dietary information, and detailed lifestyle information were called for verifying our findings.

Consistent with our findings in the association of *Lachnoclostridium*, *Alistipes,* and *Lachnospiraceae_NK4A136_group* with the different number of MetS risk factors, previous animal studies showed that the relative abundance of *Lachnoclostridium* was positively related to TG and negatively related to HDL-C in rats [45]. However, the relative abundance of *Lachnospiraceae_NK4A136_group* was negatively associated with weight gain and serum lipid levels in mice [46–48]. Additionally, the relative abundance of *Alistipes* was reduced in obese adults with metabolic diseases from China [49], obese adults from the Netherlands [50], and obese individuals with type 2 diabetes mellitus from Germany [51]. These findings suggest that the higher abundance of the genus *Lachnoclostridium* and the lower abundance of the genera *Alistipes* and *Lachnospiraceae_NK4A136_group* might contribute to the accumulation of MetS risk factors among children.

In this study, we also found that the genera *Christensenellaceae_R-7_group* and *Family_XIII_AD3011_group* performed a high ability in differentiating the two-or-more MetS risk factor group from the non-risk group. *Christensenellaceae_R-7_group* was found to be negatively related to body weight, visceral fat percentage, and FPG levels in animal experiment studies [52–54]. Moreover, it has been reported that the *Family_XIII_AD3011_group* was inversely related to glycated hemoglobin, 2 h glucose level and insulin, BMI, and secretion index in patients with type 2 diabetes mellitus, suggesting that it could be a novel predictive microbial biomarker for type 2 diabetes mellitus [55, 56]. These findings imply that the *Christensenellaceae_R-7_group* and *Family_XIII_AD3011_group* may provide a non-invasive, practical, and clinical diagnosis of the accumulation of MetS risk factors in children.

Previous studies supported our findings that the D-Glutamine and D-glutamate metabolism, as well as cysteine and methionine metabolism, play an important role in the accumulation of MetS risk factors [57–64]. It has been reported that the glutamate concentration was positively related to TG, glucose, BMI, and the increased risks of type 2 diabetes mellitus, whereas the ratio of glutamine/glutamate was negatively related to TG, glucose, BMI, and the increased risks of type 2 diabetes mellitus in Mediterranean and Spanish populations [57–59]. Furthermore, plasma total cysteine and methionine were strongly associated with MetS risk factors such as higher total cholesterol concentration, elevated BP, obesity, and type 2 diabetes mellitus [60–64]. These findings suggest that gut microbiota may be associated with the number of MetS risk factors through D-Glutamine and D-glutamate metabolism, as well as cysteine and methionine metabolism pathways.

This study has some limitations. First, the sample size in this study is smaller compared to studies in adults, so further validation with a larger sample size is warranted. However, the small sample size could still provide statistical confidence for our results because of the measurement of substantial changes in gut microbiota [65]. Second, due to the case–control study, a causal association between gut microbiota and the number of MetS risk factors cannot be concluded. Third, limited information on CRP or other cytokines and parental risk factors for MetS prohibited us from performing the effects of these clinical indicators on the association between alteration of gut microbiota and the number of MetS risk factors. Fourth, the differential gut microbiota in this study should be carefully extrapolated to children of other ethnicities, and future studies with children from other regions or countries are needed to validate our findings based on qPCR experiments. Fifth, 16S rRNA gene sequencing technology provides limited information on bacterial genes and their functions, and further research is needed to explicate the pathogenesis and mechanism of gut microbiota in children with an accumulation of MetS risk factors. Sixth, because we focused on MetS risk factors, LDL cholesterol as an important cardiovascular risk factor was not included in this study. However, we compared LDL cholesterol across the three groups and found no significant difference, partly suggesting the effect of LDL cholesterol on associations between gut microbiota and the number of MetS risk factors might be attenuated.

## Conclusions

In conclusion, we found that the community richness (i.e., the total number of species in the community) and diversity (i.e., the richness and evenness of species in the community) of gut microbiota decreased with the increased number of MetS risk factors among children. Specific gut microbiota, such as the genera *Christensenellaceae_R-7_group*, *Family_XIII_AD3011_group*, and *Lachnoclostridium*, may aid in the identification of the number of MetS risk factors among children. The disturbance of D-Glutamine and D-glutamate metabolism and cysteine and methionine metabolism pathways might contribute to the accumulation of MetS risk factors in children.

## Supplementary Information

**Additional file 1.** The methods and related software used in this study.

**Additional file 2:** **Fig. S1.** Differences in essential features of gut microbiota among the three groups. **(a)** Venn diagram. **(b)** The rarefaction curve of Shannon index. **(c)** The rarefaction curve of Sobs index. The ROC analyses for **(d)** *Ruminiclostridium_6*, **(e)*** Tyzzerella_4*, **(f)** *Ruminococcaceae_UCG-002*, **(g)** *unclassified_o__Bacteroidales*, and **(h)** *Ruminococcaceae_UCG-005*.

**Additional file 3:** **Fig. S2.** Differences in *β*-diversity of gut microbiota among the three groups. **(a)** PCoA plot based on Bray-Curtis distance matrix; **(b)** NMDS analysis.

**Additional file 4:** **Fig. S3.** Screening out differential gut microbiota biomarkers associated with numbers of MetS risk factors. **(a)** the LEfSe and LDA analyses, **(b)** the Random forest analysis, **(c)** the non-parametric Kruskal-Wallis H test.

**Additional file 5:** **Fig. S4.** Differential KEGG pathways among the three groups; Red bar, non-risk; Blue bar, one-risk; and Green bar, two-or-more risk.

**Additional file 6:** **Table S1.** The number of children with different MetS risk factors among the three groups. **Table S2.** Parental BMI and history of cardiovascular disease among the three groups. **Table S3.** Specific gut microbiota associated with MetS risk factors in children identified by LEfSe and LDA analyses. **Table S4.** The top 10 gut microbiota at the genus level screened by the random forest analysis.

## Data Availability

The datasets supporting the conclusions of this article are available in the SRA repository of the NCBI database (BioProject ID: PRJNA775883; https://www.ncbi.nlm.nih.gov/bioproject/PRJNA775883).

## Abbreviations

MetS: Metabolic syndrome
CVD: Cardiovascular diseases
BP: Blood pressure
WC: Waist circumference
FPG: Fasting plasma glucose
TG: Triglyceride
HDL-C: High-density lipoprotein cholesterol
BMI: Body mass index
gDNA: Microbial genomic DNA
PE: Pair-end
SRA: Sequence Read Archive
NCBI: The National Center for Biotechnology Information
OTU: Operational Taxonomic Units
SD: Standard deviation
ROC: Receiver operating characteristic curve
AUC: Area under the ROC curve
FDR: False discovery rate
PCoA: The Principal Coordinate Analysis
NMDS: Non-metric multidimensional scaling analysis
LEfSe: The Linear Discriminant Analysis Effect Size
LDA: The linear discriminant analysis
KEGG: The Kyoto Encyclopedia of Genes and Genomes
CAMP: Cationic antimicrobial peptide
