# Supplementary material for: The number of metabolic syndrome risk factors predicts alterations in gut microbiota in Chinese children from the Huantai study
Source: BMC Pediatr. 2023 Apr 21;23:191. doi: 10.1186/s12887-023-04017-x (PMC10120097; doi:10.1186/s12887-023-04017-x)
Supplement: Supplementary file 1 — Additional file 1. The methods and related software used in this study. [file 12887_2023_4017_MOESM1_ESM.doc]

The methods and related software we used in this study:

1. The rarefaction curve:

Mothur (version v.1.30.2; https://mothur.org/wiki/calculators/) was used to calculate *α*-diversity index under different random sampling, and R language (Version 3.3.1) was used to make graphs.

2. The Venn diagram analysis:

R language (Version 3.3.1).

3. The bar plot:

R language (Version 3.3.1).

4. *α*-diversity:

mothur (version v.1.30.2; https://mothur.org/wiki/calculators/) was used to calculate *α*-diversity index.

5. Principal Coordinate Analysis (PCoA):

Vegan package in R language (Version 3.3.1)

6. Non-metric Multidimensional scaling (NMDS) analyses:

Vegan package in R language (Version 3.3.1)

7. The non-parametric Kruskal-Wallis H test:

Stats package in R language and scipy package in Python.

8. The Linear discriminant analysis effect size (LEfSe) and the linear discriminant analysis (LDA):

LEfSe (<http://huttenhower.sph.harvard.edu/galaxy/root?tool_id=lefse_upload>) software.

9. The random forest model analysis:

randomForest package in R language (Version 3.3.1).

10. The Kyoto Encyclopedia of Genes and Genomes (KEGG):

Phylogenetic Investigation of Communities by Reconstruction of Unobserved States 2 (PICRUSt2) software.

11. The receiver operating characteristic (ROC) analysis

pROC package in R language (Version 4.1.0).
