# Supplementary figures and images for: The number of metabolic syndrome risk factors predicts alterations in gut microbiota in Chinese children from the Huantai study
Source: BMC Pediatr. 2023 Apr 21;23:191. doi: 10.1186/s12887-023-04017-x (PMC10120097; doi:10.1186/s12887-023-04017-x)

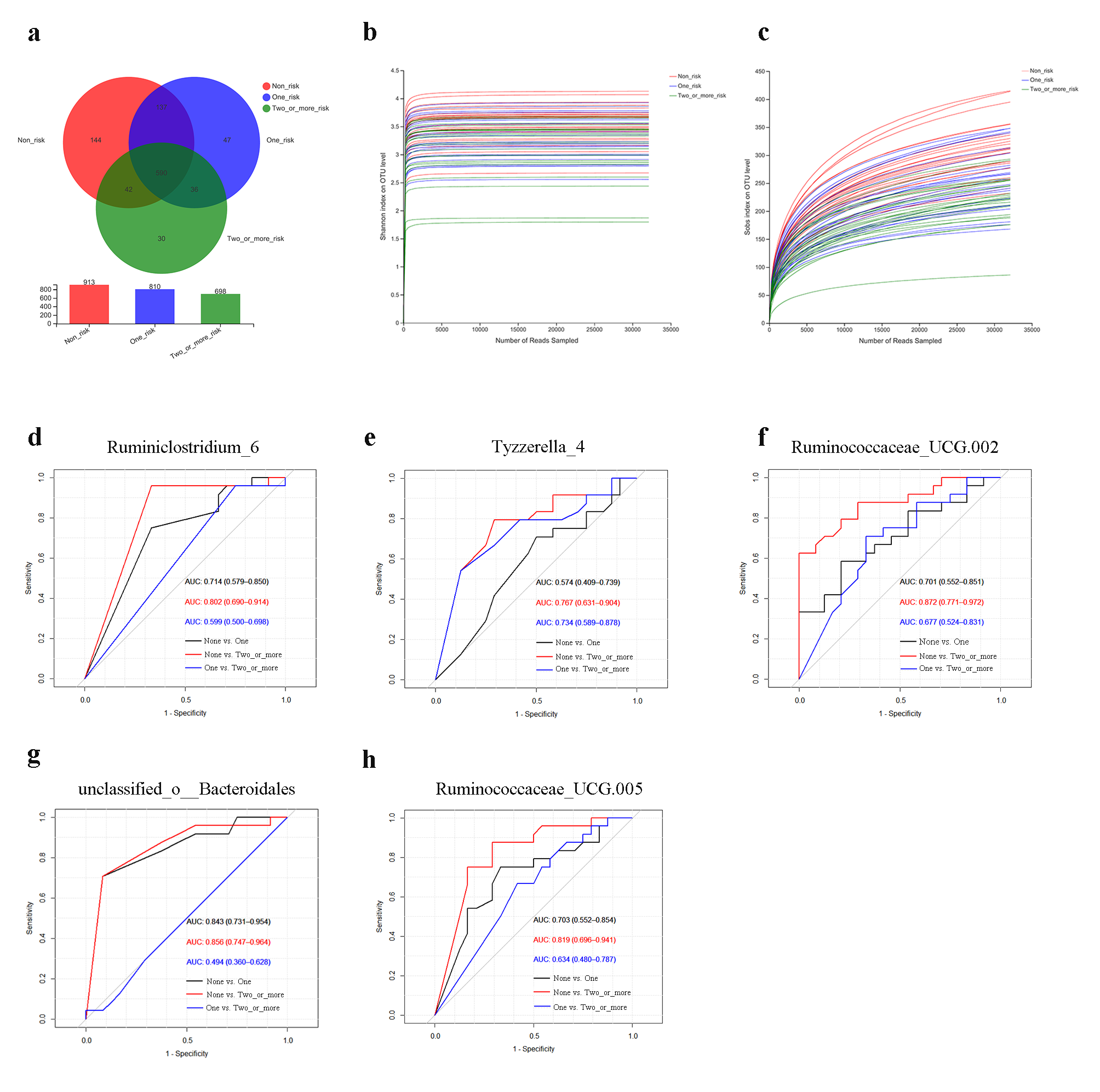

Supplement: Supplementary file 2 — Additional file 2: Fig. S1. Differences in essential features of gut microbiota among the three groups. (a) Venn diagram. (b) The rarefaction curve of Shannon index. (c) The rarefaction curve of Sobs index. The ROC analyses for (d) Ruminiclostridium_6, (e) Tyzzerella_4, (f) Ruminococcaceae_UCG-002, (g) unclassified_o__Bacteroidales, and (h) Ruminococcaceae_UCG-005. [file 12887_2023_4017_MOESM2_ESM.tif]

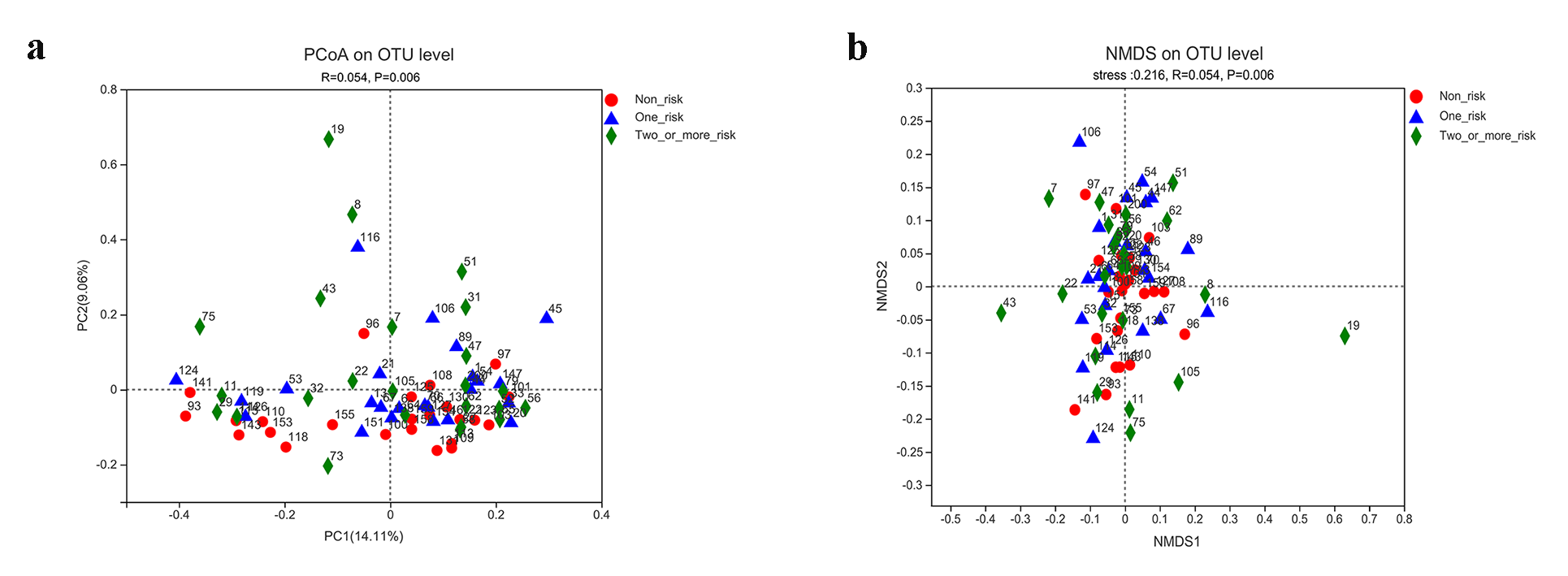

Supplement: Supplementary file 3 — Additional file 3: Fig. S2. Differences in β-diversity of gut microbiota among the three groups. (a) PCoA plot based on Bray-Curtis distance matrix; (b) NMDS analysis. [file 12887_2023_4017_MOESM3_ESM.tif]

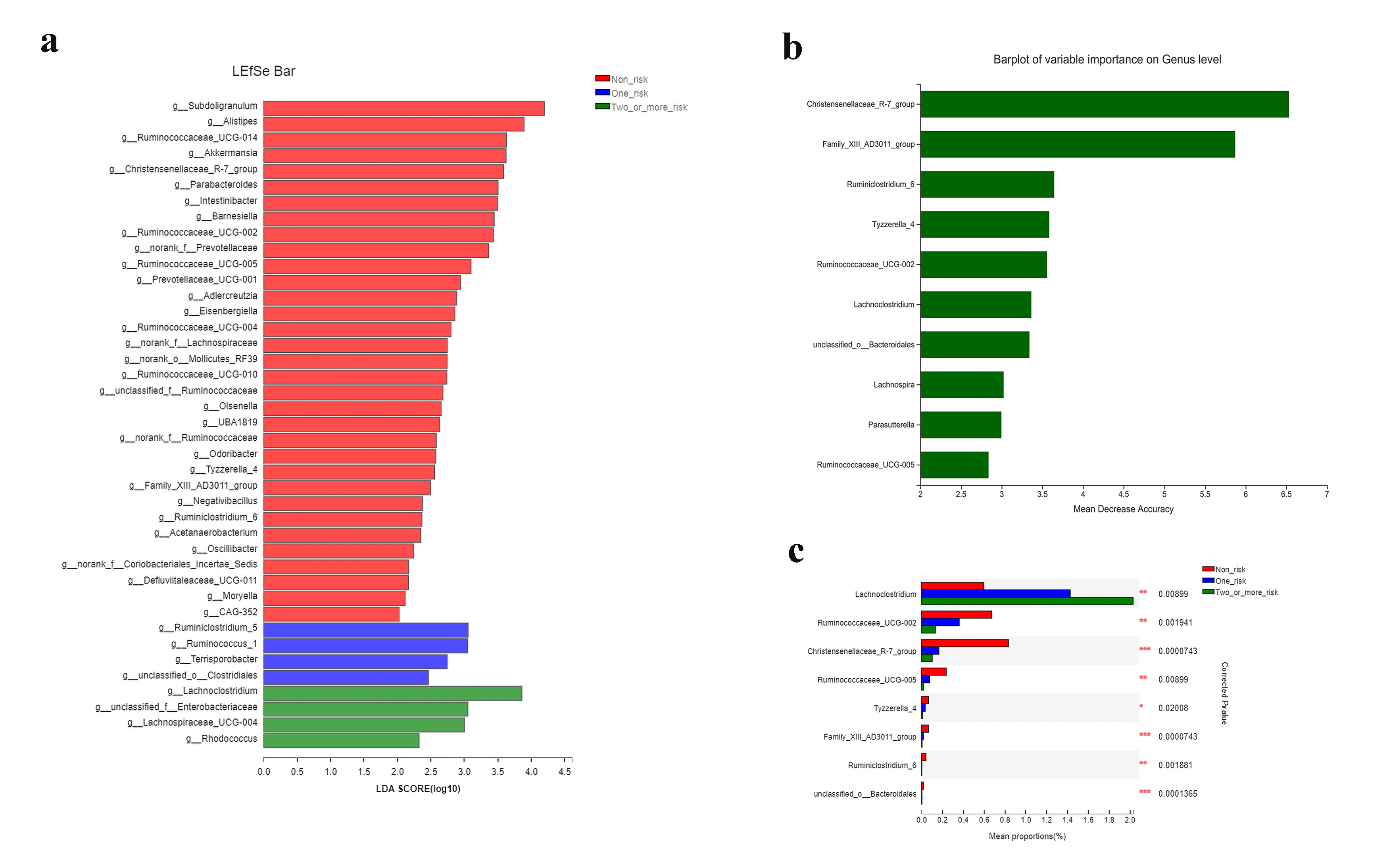

Supplement: Supplementary file 4 — Additional file 4: Fig. S3. Screening out differential gut microbiota biomarkers associated with numbers of MetS risk factors. (a) the LEfSe and LDA analyses, (b) the Random forest analysis, (c) the non-parametric Kruskal-Wallis H test. [file 12887_2023_4017_MOESM4_ESM.tif]
