# Supplementary material for: The number of metabolic syndrome risk factors predicts alterations in gut microbiota in Chinese children from the Huantai study
Source: BMC Pediatr. 2023 Apr 21;23:191. doi: 10.1186/s12887-023-04017-x (PMC10120097; doi:10.1186/s12887-023-04017-x)
Supplement: Supplementary file 6 — Additional file 6: Table S1. The number of children with different MetS risk factors among the three groups. Table S2. Parental BMI and history of cardiovascular disease among the three groups. Table S3. Specific gut microbiota associated with MetS risk factors in children identified by LEfSe and LDA analyses. Table S4. The top 10 gut microbiota at the genus level screened by the random forest analysis. [file 12887_2023_4017_MOESM6_ESM.doc]

| **Table S1** The number of children with different MetS risk factors among the three groups | | | |  |
| --- | --- | --- | --- | --- |
| **MetS risk factors** | **Non-risk (n=24)** | **One-risk (n=24)** | **Two-or-more-risks (n=24)** |  |
|  |
| Abdominal obesity | 0 | 12 | 22 |  |
| High BP | 0 | 8 | 17 |  |
| High FPG | 0 | 2 | 4 |  |
| High TG | 0 | 2 | 9 |  |
| Low HDL-C | 0 | 0 | 6 |  |
| Abbreviations: MetS: metabolic syndrome; BP: blood pressure; FPG: fasting plasma glucose; TG: triglyceride; HDL-C: high-density lipoprotein cholesterol. | | | |  |

| Table S2 Parental BMI and history of cardiovascular disease among the three groups | | | | | |
| --- | --- | --- | --- | --- | --- |
| **Family history** | **Non-risk**  **(n=24)** | **One-risk**  **(n=24)** | **Two-or-more-risks**  **(n=24)** | ***F/χ^2^*** | P-value |
| BMI of father (kg/m^2^; mean ± sd) | 24.31±5.47 | 24.96±2.28 | 25.33±3.92 | 1.848^a^ | 0.165 |
| BMI of mother (kg/m^2^; mean ± sd) | 22.11±1.96 | 23.53±3.33 | 23.77±2.98 | 2.260^a^ | 0.112 |
| Hypertension of father (n %) |  |  |  | 1.122^b^ | 0.865 |
| No | 21 (87.5%) | 23 (95.8%) | 22 (91.7%) |  |  |
| Yes | 3 (12.5%) | 1 (4.2%) | 2 (8.3%) |  |  |
| Hypertension of mother (n %) |  |  |  | —^c^ | — |
| No | 24 (100.0%) | 24 (100.0%) | 24 (100.0%) |  |  |
| Yes | 0 (0.0%) | 0 (0.0%) | 0 (0.0%) |  |  |
| Heart disease of father (n %) |  |  |  | —^c^ | — |
| No | 24 (100.0%) | 24 (100.0%) | 24 (100.0%) |  |  |
| Yes | 0 (0.0%) | 0 (0.0%) | 0 (0.0%) |  |  |
| Heart disease of mother (n %) |  |  |  | 1.85^b^ | 1.000 |
| No | 23 (95.8%) | 24 (100.0%) | 24 (100.0%) |  |  |
| Yes | 1 (4.2%) | 0 (0.0%) | 0 (0.0%) |  |  |
| Stroke of father (n %) |  |  |  | —^c^ | — |
| No | 24 (100.0%) | 24 (100.0%) | 24 (100.0%) |  |  |
| Yes | 0 (0.0%) | 0 (0.0%) | 0 (0.0%) |  |  |
| Stroke of mother (n %) |  |  |  | —^c^ | — |
| No | 24 (100.0%) | 24 (100.0%) | 24 (100.0%) |  |  |
| Yes | 0 (0.0%) | 0 (0.0%) | 0 (0.0%) |  |  |
| Diabetes of father (n %) |  |  |  | 2.74^b^ | 0.324 |
| No | 22 (91.7%) | 24 (100.0%) | 24 (100.0%) |  |  |
| Yes | 2 (8.3%) | 0 (0.0%) | 0 (0.0%) |  |  |
| Diabetes of mother (n %) |  |  |  | —^c^ | — |
| No | 24 (100.0%) | 24 (100.0%) | 24 (100.0%) |  |  |
| Yes | 0 (0.0%) | 0 (0.0%) | 0 (0.0%) |  |  |

Note: BMI: body mass index; sd, standard deviation; ^a^ Analysis of Variance; ^b^ Fisher’s exact test; ^c^ None of the parents in the three groups had a history of cardiovascular disease so no statistical test was required.

| **Table S3** Specific gut microbiota associated with MetS risk factors in children identified by LEfSe and LDA analyses | | | |
| --- | --- | --- | --- |
| **Species name** | **Groups** | **LDA_value** | ***P_*value** |
| *Subdoligranulum* | Non-risk | 4.20 | 0.026 |
| *Alistipes* | Non-risk | 3.89 | <0.001 |
| *Ruminococcaceae_UCG-014* | Non-risk | 3.63 | 0.002 |
| *Akkermansia* | Non-risk | 3.62 | 0.001 |
| *Christensenellaceae_R-7_group* | Non-risk | 3.58 | <0.001 |
| *Parabacteroides* | Non-risk | 3.50 | 0.036 |
| *Intestinibacter* | Non-risk | 3.50 | 0.002 |
| *Barnesiella* | Non-risk | 3.45 | 0.004 |
| *Ruminococcaceae_UCG-002* | Non-risk | 3.43 | <0.001 |
| *norank_f__Prevotellaceae* | Non-risk | 3.37 | 0.046 |
| *Ruminococcaceae_UCG-005* | Non-risk | 3.10 | <0.001 |
| *Prevotellaceae_UCG-001* | Non-risk | 2.94 | 0.046 |
| *Adlercreutzia* | Non-risk | 2.88 | 0.018 |
| *Eisenbergiella* | Non-risk | 2.86 | 0.024 |
| *Ruminococcaceae_UCG-004* | Non-risk | 2.80 | <0.001 |
| *norank_f__Lachnospiraceae* | Non-risk | 2.74 | 0.031 |
| *norank_o__Mollicutes_RF39* | Non-risk | 2.74 | 0.013 |
| *Ruminococcaceae_UCG-010* | Non-risk | 2.74 | <0.001 |
| *unclassified_f__Ruminococcaceae* | Non-risk | 2.68 | 0.014 |
| *Olsenella* | Non-risk | 2.65 | 0.026 |
| *UBA1819* | Non-risk | 2.63 | 0.004 |
| *norank_f__Ruminococcaceae* | Non-risk | 2.58 | <0.001 |
| *Odoribacter* | Non-risk | 2.57 | 0.001 |
| *Tyzzerella_4* | Non-risk | 2.56 | 0.002 |
| *Family_XIII_AD3011_group* | Non-risk | 2.49 | <0.001 |
| *Negativibacillus* | Non-risk | 2.38 | 0.017 |
| *Ruminiclostridium_6* | Non-risk | 2.37 | <0.001 |
| *Acetanaerobacterium* | Non-risk | 2.35 | 0.012 |
| *Oscillibacter* | Non-risk | 2.24 | 0.036 |
| *norank_f__Coriobacteriales_Incertae_Sedis* | Non-risk | 2.17 | 0.001 |
| *Defluviitaleaceae_UCG-011* | Non-risk | 2.17 | <0.001 |
| *Moryella* | Non-risk | 2.11 | 0.007 |
| *CAG-352* | Non-risk | 2.02 | 0.005 |
| *Ruminiclostridium_5* | One-risk | 3.06 | 0.001 |
| *Ruminococcus_1* | One-risk | 3.05 | 0.005 |
| *Terrisporobacter* | One-risk | 2.74 | 0.017 |
| *unclassified_o__Clostridiales* | One-risk | 2.46 | 0.014 |
| *Lachnoclostridium* | Two-or-more-risk | 3.86 | 0.001 |
| *unclassified_f__Enterobacteriaceae* | Two-or-more-risk | 3.05 | 0.043 |
| *Lachnospiraceae_UCG-004* | Two-or-more-risk | 3.00 | 0.001 |
| *Rhodococcus* | Two-or-more-risk | 2.32 | 0.046 |

| **Table S4** The top 10 gut microbiota at the genus level screened by the random forest analysis | | | |
| --- | --- | --- | --- |
| **Feature** | **Importance** | **Level** |  |
| *Christensenellaceae_R-7_group* | 6.53 | genus |  |
| *Family_XIII_AD3011_group* | 5.87 | genus |  |
| *Ruminiclostridium_6* | 3.65 | genus |  |
| *Tyzzerella_4* | 3.59 | genus |  |
| *Ruminococcaceae_UCG-002* | 3.56 | genus |  |
| *Lachnoclostridium* | 3.37 | genus |  |
| *unclassified_o__Bacteroidales* | 3.34 | genus |  |
| *Lachnospira* | 3.02 | genus |  |
| *Parasutterella* | 3.00 | genus |  |
| *Ruminococcaceae_UCG-005* | 2.84 | genus |  |
